# Isolating the acute metabolic effects of carbohydrate restriction on postprandial metabolism with or without energy restriction: a crossover study

**DOI:** 10.1007/s00394-025-03646-5

**Published:** 2025-03-20

**Authors:** Hayriye Biyikoglu, M. Denise Robertson, Adam L. Collins

**Affiliations:** 1 Department of Nutritional Sciences, Faculty of Health and Medical Sciences, University of Surrey, Surrey, GU2 7XH UK; 2 School of Life and Health Sciences, University of Roehampton, London, SW15 5PH UK

**Keywords:** Low-carbohydrate, Intermittent energy restriction, Postprandial, Cross-over

## Abstract

Low-carbohydrate diets and intermittent energy restriction may offer metabolic advantages in fuel utilisation, that are independent of weight loss. The underlying mechanisms for these effects are unclear but may involve extensions of the catabolic phase and/or attenuation of insulin secretion. To address this gap, we aimed to investigate the independent acute metabolic effect of carbohydrate restriction at varying energy levels. Twelve, (six female) healthy overweight/obese participants (27.3 ± 1.8 years; 25.2 ± 1.6 kg/m^2^) completed this three-way study. Volunteers followed three diets for one day (36 h, covering the intervention day and overnight fasting), separated by 5-day washout: a normal carbohydrate, energy-balanced diet (nEB, 55% CHO), a low-carbohydrate, energy-balanced diet (LCEB, 50 g/day CHO), and a low-carbohydrate, energy-restricted diet (LC25, 50 g/day CHO with 75% energy restriction). Fasting and serial postprandial (360 min) measurements to a mixed test meal were collected the following morning. Additionally, subjective appetite responses and two-day subsequent *ad libitum* food intake was assessed. Both low-carbohydrate with and without energy restriction diets induced comparable decrease in triacylglycerol iAUC (*p* = 0.02, *p* = 0.04, respectively), and respiratory quotient (both *p* < 0.01) along with increase in non-esterified fatty acids (both *p* < 0.01) and 3-hydroxybutyrate (*p* = 0.001, *p* = 0.01, respectively) levels. Compared to a non-restricted carbohydrate, energy-balanced diet, postprandial glucose levels significantly increased in the LCEB arm (*p* = 0.024) and showed a rising trend in the LC25 arm (*p* = 0.07). Neither insulin responses nor resting, and diet-induced thermogenesis were significantly altered by variations in energy or carbohydrate content. These findings demonstrate that carbohydrate restriction, without altering energy intake, can elicit effects similar to those observed in short-term fasting. As such we propose a strategy of repeated carbohydrate restriction cycles alone may be an emerging alternative approach for the enhancement of cardiometabolic health, warranting further investigation.

**Supplementary Information:**

The online version contains supplementary material available at 10.1007/s00394-025-03646-5.

## Introduction

Having excess body weight correlates significantly with a rise in comorbidities, such as cardiovascular disease (CVD), cancer, and type 2 diabetes mellitus (T2DM), as corroborated by numerous epidemiological studies [1] and imposes considerable demands on healthcare systems [2, 3]. Paradoxically, elevated cardio-metabolic risk can also manifest in individuals of a normal weight [4], whilst some with higher body weight may maintain metabolic health [5]. This contrast highlights the complexity of metabolic health beyond mere weight status, emphasizing the critical need for dietary interventions to not only manage weight but also comprehensively mitigate cardio-metabolic risks. continuous energy restriction (CER) has traditionally been the primary means of tackling excess body weight issues, but more recently intermittent energy restriction (IER) has, emerged as an effective alternative for weight loss and the regulation of cardio-metabolic disease markers [6, 7]. The appeal of IER is that individuals consume a typical diet for five days with substantial caloric restriction (25% of the recommended daily energy intake) on only two non-consecutive days per week. Meta-analyses reveal that IER is as effective as CER in enhancing various health markers, including reductions in fasted triacylglycerol (TAG), LDL-C, HBA1c, and metrics of body composition, and increased HDL-C levels [8–10]. However, research on dietary consumption patterns indicates despite a higher initial adherence to IER compared to CER, compliance declines over time [11, 12]. This suggests a need for alternative dietary strategies. It is documented that a 5-10% weight loss can enhance glycaemic and lipid profiles [13], yet while both CER and IER diets facilitate comparable weight loss, the unique attributes of IER may exert additional benefits. In particular, Antoni et al. [14] demonstrated that even with matched weight loss, IER may confer superior benefits on postprandial lipaemia compared to CER diets. This advantage is possibly attributable to the repeated metabolic shifts from glucose to fatty acid and ketone utilisation induced by IER regimens [15]. This is akin to improved “metabolic flexibility”, defined as a capacity to efficiently switch between fat oxidation and other metabolic processes depending on fuel availability and metabolic demand [16] and could be a key factor underlying the benefits of IER. More relevantly, these metabolic shifts can be initiated by reduced carbohydrate alone rather than by a negative energy balance per se [17]. Given inherently low carbohydrate content on restricted days of IER diets, appreciating the role of carbohydrate content becomes increasingly pertinent. A recent cross-over study assessing the postprandial metabolic health markers following various levels of energy restriction (ER) for one day found that both partial and total ER altered fasting and postprandial metabolic responses, with these changes indicative of a dose-responsive pattern in relation to the ER level [14]. The authors attributed this effect to the varying levels of carbohydrate availability in partial ER. Such an observation underscores the complex role of carbohydrate intake in shaping metabolic responses under varying degrees of energy restriction and questions the extent to which these responses might be attributed solely to variations in carbohydrate intake. Our study aims to investigate the acute metabolic effects of carbohydrate restriction at varying energy levels, to isolate and examine the specific metabolic responses exclusively linked to carbohydrate reduction. The study secondary outcomes involve examining the subsequent adjustments in energy consumption and the energy expenditure (i.e., energy balance), which might influence the long-term effectiveness of diets for weight management.

## Methods

### Participants and recruitment

Participants were aged 20–65 years and classified as overweight or obese (BMI < 25 kg/m^2^) and were recruited from the University of Surrey (UK) and the wider community through posters and email advertisements. Participants were weight stable ( < ± 3 kg in the last three months), with no significant medical history. Body composition was assessed via bioelectrical impedance scale (Tanita, Japan). Screening excluded individuals on medications impacting postprandial glucose or lipid responses, including insulin and thyroid therapy, with additional exclusions for beta-blockers, proton pump inhibitors, hormonal therapy, and anxiety medications, assessed case by case. Further exclusions included exercising more than three times per week (due to potential BMI inaccuracies from increased muscle mass), pregnancy or breastfeeding, excessive caffeine or alcohol intake, current eating or psychiatric disorders, and adherence to strict diets like veganism. All recruited female participants were either postmenopausal, used hormonal contraceptives or were in the follicular phase of their menstrual cycle to account for potential menstrual cycle-related effects between visits. The study obtained a favourable ethical opinion from the University of Surrey ethics committee (UEC 2019 008 FHMS) and was carried out in compliance with the principles outlined in the Declaration of Helsinki. The study is registered at clinicaltrials.gov: NCT06387940.

### Sample size

A comparable cross-over study demonstrated that a sample size of 10 participants was sufficient to achieve a statistical power of 90%. This power aimed to detect a mean differential of 89 units (SEM 13) in the postprandial TAG iAUC values, our primary outcome, between a one-day 75% energy-restricted diet and a 100% energy-balanced diet [18]. Upon retrospective analysis, it was determined that with 12 participants, this study achieved 90% power at a two-sided significance level of 0.05, to detect a mean difference of 57 units (SD = 54.6) between the intervention arms (nEB vs. LC25).

### Study design

This was a randomised, three-way crossover design, in which participants followed, three, one-day (36 h) dietary interventions in a random order, with a 5-day washout period between interventions (Supplementary Fig.). An energy-balanced diet (EB) served as the control, in comparison to a low-carbohydrate energy-balanced diet (LCEB), and a low-carbohydrate energy-restricted diet (LC25). After completing each diet day, participants attended a metabolic study day to assess appetite and measure blood metabolites in response to a standard meal challenge. Participants documented their dietary intake from the ad libitum meal served during the laboratory visit until midnight the following day to evaluate short-term energy compensation.

### Dietary interventions (Day 1)

Participants recorded their usual dietary intake over a three-day period before the study commencement. The lead-in period included a standardised pre-intervention dinner and a minimum 5-day washout between treatments, during which participants maintained their habitual dietary intake. All food items for dietary intervention days, including the lead-in dinner, were prepared and personally supplied to participants. Participants were assigned to one of three” calorie groups” based on their daily energy requirements, estimated using the Henry predictive equation and their physical activity levels, and calculated separately for males and females (Table 1). Protein in the low-carbohydrate diets was capped at 0.8 g/kg body weight, aligning with the Recommended Dietary Allowance for healthy adults, to attribute effects to carbohydrate and energy changes, avoiding confounding influences of protein’s satiety, insulinotropic effects [19] and thermic effect [20]. To maintain unbiased appetite measurements and minimize impacts on digestive and metabolic systems, the study’s menu kept a consistent visual appearance. Meal compositions remained the same, adjusting only portion sizes for different calorie groups. For low-carbohydrate diets, visually similar substitutes like cauliflower rice for rice and courgette for spaghetti were used.

#### Energy-balanced diet (nEB)

Diet served as control, included 100% of their estimated isoenergetic needs adhering to the principles of the Eatwell Guide [21]. Carbohydrates comprised 55% of total caloric intake.

#### Low-carbohydrate energy-balanced diet (LCEB)

Diet included only 50 g of carbohydrate yet provided 100% of their estimated isoenergetic needs.

#### Low-carbohydrate energy-restricted diet (LC25)

Diet included only 50 g of carbohydrate and provided 25% of estimated isoenergetic needs.

**Table 1:**
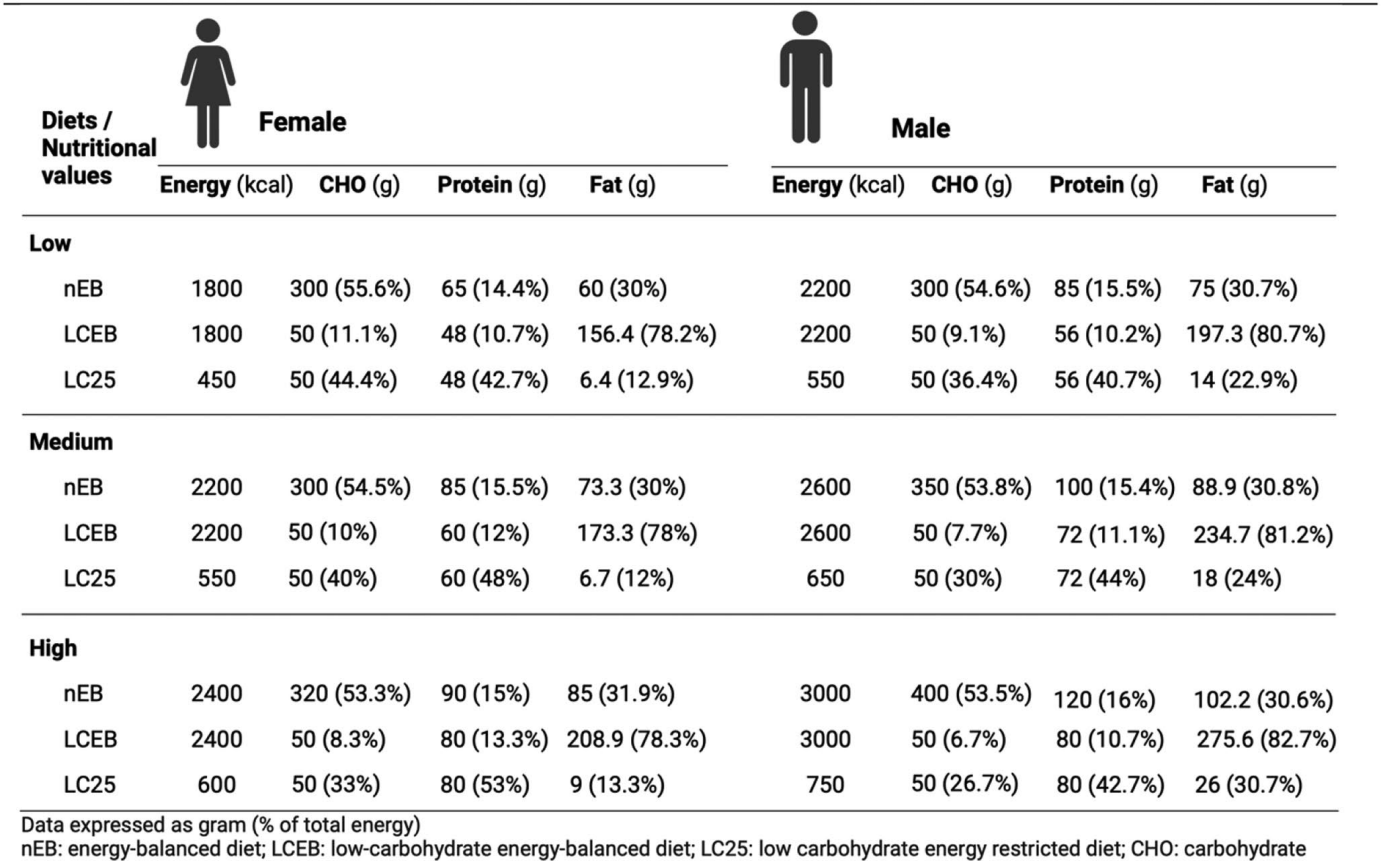
Energy and macronutrient composition of the intervention diets according to low, medium, and high calorie groups

### Laboratory visits (Day 2)

On designated diet days, participants were instructed to complete their standardised meal by 20:00 h and maintain sufficient intake of non-caloric fluids while refraining from alcohol consumption and vigorous exercise. Following each diet day, participants arrived at the Clinical Investigation Unit of the University of Surrey around 8:00 h after fasting overnight with only water allowed. After a 30-minute rest period, resting energy expenditure (REE) and substrate utilisation (respiratory quotient [RQ]) were assessed using indirect calorimetry (GEM Nutrition). Following this assessment, an indwelling cannula was inserted, and fasted blood samples were collected. To evaluate fasting appetite sensation, validated paper-based visual analogue scales (VAS) were utilized. Then, participants were provided with a liquid test meal (400 ml Fortisip, 40 ml Calogen Extra Shot; Nutricia) to be consumed within 5 min. This meal was designed to mimic the energy composition of full English breakfast (705 kcal, Nutritics; Ireland) and included 760 kcal, 75.4 g carbohydrates (39% of total energy), 28.6 g protein (15% of total energy), and 39.3 g fat (46% of total energy). The use of this standardised meal aimed to minimize potential variations in postprandial measurements attributed to food preparation and digestive factors. Following the test meal, multiple postprandial measurements of blood metabolites, energy expenditure, substrate utilization, and appetite measures were taken at regular intervals over the next 360 min. The study concluded with a pre-weighed ad-libitum pasta meal (2378 kcal; 50% carbohydrate, 20% protein, 30% fat), served within 10 min of the final measurement, allowing participants to eat until full. Intake was recorded based on consumption. Participants then returned to their normal diets, logging their food intake in diaries until midnight the following day.

#### Blood biochemistry

Blood samples were collected at the baseline measurement (pre-meal) and subsequently at regular intervals of 15, 30, 60, 90, 120, 180, 240, 300, and 360 min into EDTA (for TAG, NEFA, insulin, 3-*β*-hydroxybutyrate (3-OHB) and glucagon-like peptide 1 (GLP-1) analysis) and sodium oxalate (for plasma glucose analysis). 200 kallikrein inhibiting units of aprotinin per ml of whole blood was added to EDTA tubes for the GLP-1 analysis. Samples were then centrifuged (1620 g; 10 min; 4 °C) and separated. Plasma aliquots were then stored at -20 °C (for glucose, lipids and 3-OHB) and − 80 °C (for GLP-1 and insulin), prior to analysis.

Plasma glucose, TAG, NEFA, and 3-OHB concentrations were quantified using an automated photometric Indiko™ clinical chemical analyser (Thermo Fisher Scientific, United States). Insulin and GLP-1 levels were determined through an enzyme-linked immunosorbent assay (ELISA, Millipore). The analysis of samples were performed in batches, thereby ensuring that samples from each individual were assessed within the same assay, minimizing potential inter-assay variability.

#### Assessment of insulin sensitivity

Matsuda Index [22] and HOMA-IR [23] measured insulin sensitivity, while Insulinogenic Index [24] and Disposition Index [25] evaluated β-cell function. M (Stumvoll) and first- and second-phase insulin indices [26] estimated postprandial insulin response and secretion.

#### Indirect calorimetry

REE and substrate utilisation were assessed using indirect calorimetry (GEM Nutrition Ltd, UK). During the measurements, participants maintained a semi-supine position and wore a ventilated Perspex hood, enabling a continuous air flow for the analysis of the relative concentrations of oxygen and carbon dioxide in both inspired and expired air. To ensure accurate measurement, calibrations were conducted before the start of each testing day, and each participant was also measured repeatedly on the same machine. The Weir equation [27] was employed for REE analysis, while substrate utilisation was represented as RQ, (calculated as VCO_2_*/*VO_2_).Fasting and postprandial substrate oxidation rates of fat and carbon dioxide were determined using Frayn’s non-protein stoichiometric equations [28]. Diet-induced thermogenesis (DIT) was calculated by subtracting REE from postprandial energy expenditure [29].

#### Dietary analyses

Dietary data from participants were recorded in food diaries and analysed with Nutritics software (Nutritics Software, Ireland). These diaries included instructions and visuals to help estimate portion sizes. Dietary intake was logged for three days before the study (two weekdays and one weekend) and from the ad libitum meal during the laboratory visit until midnight the next day.

The impact of a single day’s diet on eating habits was assessed by comparing baseline intake to post-diet day data.

#### Visual analogue scales

Participant’s perceived appetite sensations were measured using validated visual analogue scales (VAS) [30]. These 100-mm scales had endpoints representing extreme hunger states. Participants evaluated eight variables including satiety, desire to eat, food volume capacity, thirst, and cravings for sugary, fatty, salty, and savoury foods. They marked their perceived appetite levels every two hours on diet days and at blood measurement intervals on study day.

#### Statistical analyses

Incremental and decremental area under the curve (iAUC, dAUC, respectively) for metabolites were calculated using Simpson’s rule [31], implemented in Python 3.8 (Python Software Foundation). All statistical analyses were conducted using GraphPad Prism 9 (GraphPad Software Inc.) Owing to the sample size of data, non-parametric testing was identified as the most appropriate method for conducting the statistical analysis in this study. The Friedman test was used to examine differences among the three experimental groups, complemented by Dunn’s post hoc testing for multiple pairwise comparisons. The Wilcoxon signed-rank test was employed for paired comparisons. For time-course data, repeated measures of ANOVA, along with Tukey’s post hoc testing, was used. Statistical significance was established at *p* < 0.05. All data are presented as mean ± SEM.

## Results

### Participant characteristics

Initially, 20 participants (12 females), aged 20–65 years, consented; however, eight participants (6 females) did not complete due to non-compliance (*n* = 4), cannulation issues (*n* = 2), or Covid-19 pandemic-related lab closure. Consequently, the final sample consisted of twelve completers (Table 2).

**Table 2:** Participant characteristics

	All Participants (*n* = 12)	Female (*n* = 6)	Male (*n* = 6)
Age (years)	27.3 ± 1.8	24 ± 1.0	30.7 ± 3.0
Body weight (kg)*	75.7 ± 5.6	64.5 ± 7.8	86.9 ± 5.0
BMI (kg/m^2^)*	25.2 ± 1.6	23.5 ± 3.9	26.9 ± 1.0
% body fat*	28.1 ± 0.6	33.1 ± 1.2	23 ± 1.7

Mean ± SEM

*significant difference between sexes (*p* < 0.005), unpaired t-test

### Postprandial metabolic responses

#### TAG responses

**Fig. 1:**
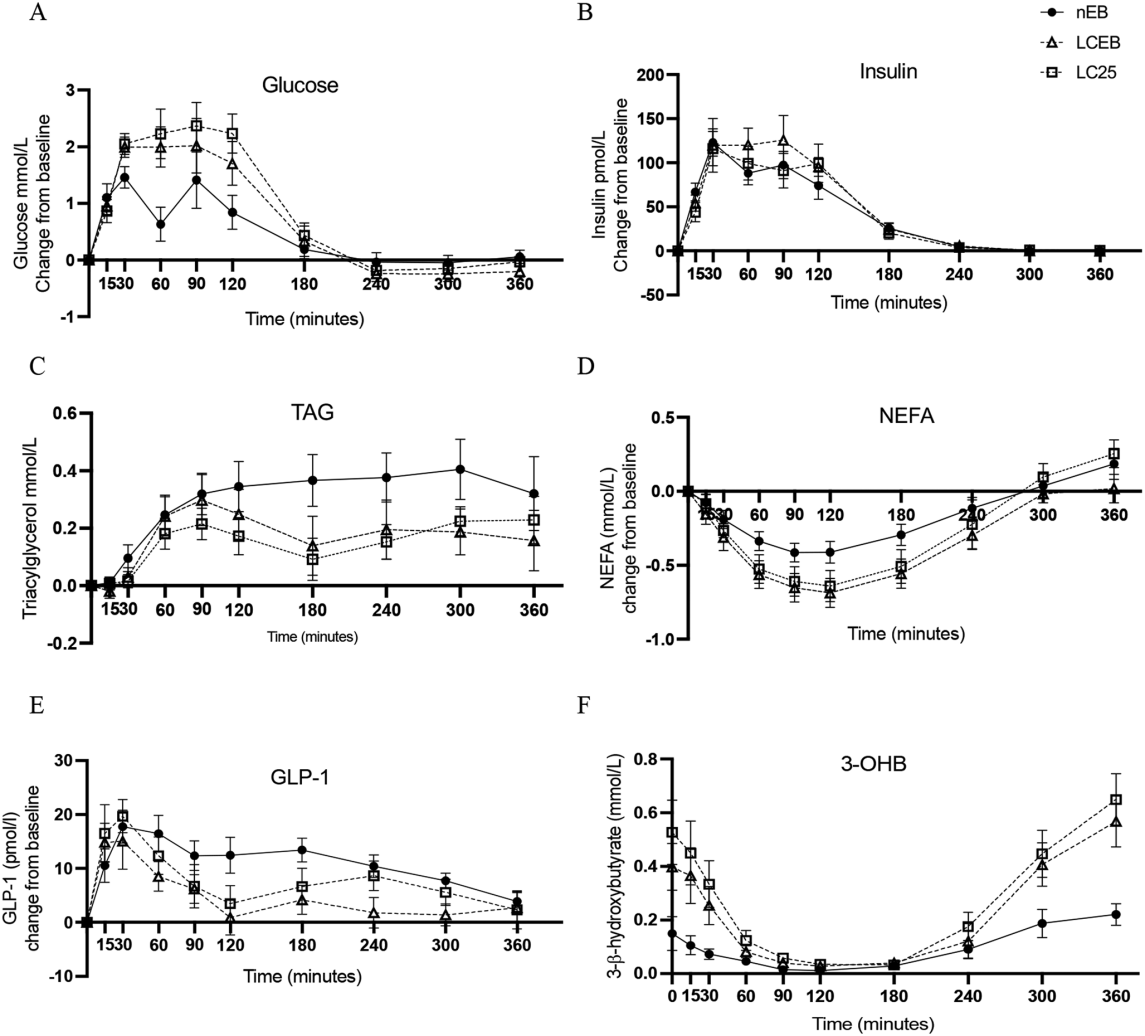
Postprandial substrate responses to liquid test meal. Data presented with standard error bars Glucose, Insulin, TAG, NEFA: *n 12*, GLP-1:*n 8*

A statistically significant main effect of diet was observed on postprandial TAG levels across the intervention arms (*p* = 0.02), with significant differences between both LC diets and nEB (both p *<* 0.05) (Fig. 1(C)). The postprandial TAG iAUC values were significantly lower by 61.17 (SEM 18.86) mmol/L·min, for LCEB (*p* = 0.04) and by 81.78 (SEM 12.53) mmol/L·min for LC25 (*p* = 0.02) compared to nEB. However, iAUC comparisons between the two LC arms showed no statistical significance (p *>* 0.99).

### NEFA and 3-OHB responses

As expected, both postprandial plasma NEFA (Fig. 1(D)) and 3-OHB levels (Fig. 1 (F)) decreased until approximately T120 and then steadily increased until T360 across all study arms. The two LC arms displayed similar trends in these markers. A significant main effect of the intervention (p *<* 0.001) and a diet x time interaction (*p* = 0.002) were noted in postprandial NEFA concentrations, with both LC arms showing statistically significant differences from the nEB arm (p *<* 0.001). Although decremental area under the curve (dAUC) for NEFA was not significantly different between the intervention arms. Similar to NEFA, significant effects of diet (p *<* 0.001) and diet x time interaction (*p* = 0.004) were observed in postprandial 3-OHB concentrations. Post-hoc analysis revealed significant differences between LCEB and LC25 compared to nEB (*p* < 0.001). However, the AUC (dAUC) of 3-OHB differed significantly only between nEB (-22.48 ± 19.96 mmol/L·min) and LC25 (-106.5 ± 33.40 96 mmol/L·min), with LCEB (-74.46 ± 22.01 96 mmol/L·min) showing no significant differences.

### Glucose and insulin response

Following the nEB arm, postprandial glucose changes exhibited a bimodal pattern with peaks at T30 and T90, returning to baseline Fig. 1(A). In contrast, both LC arms displayed an initial glucose increase, peaking at T30 and remaining elevated until T90 before reverting to baseline. Statistically significant interactions between time x diet were observed in postprandial plasma glucose responses (*p* = 0.0016), though no significant differences were found between diet arms in post hoc comparisons (p *>* 0.05). The glucose iAUC was higher in both LC arms: LCEB recorded 305 (SEM 40.36) mmol/min per L and LC25 at 337.3 (SEM 40.27) mmol/min per L, compared to 202.2 (SEM 40.46) mmol/min per L for nEB. LCEB differed significantly from nEB (*p* = 0.03), while LC25 showed a non-significant trend (*p* = 0.07). The postprandial insulin response showed no significant differences across diets (Fig. 1(B)). No significant differences were observed in insulin sensitivity (Matsuda Index, HOMA-IR, M Stumvoll), or β-cell function (Insulinogenic Index, Disposition Index, first- and second-phase insulin) between diet conditions.

### GLP-1 responses

Postprandial GLP-1 responses exhibited non-significant trends for both diet main effect and diet-by-intervention interaction (*p* = 0.07 for both), suggestive of potentially lower GLP-1 levels in LCEB and LC25 interventions (Fig. 1(E)). However, post hoc analysis, conducted to explore this trend (*p* < 0.1) and account for potential sample size limitations, showed significant differences between LCEB and nEB (*p* = 0.01) and between LC25 and nEB (*p* = 0.0004). Similarly, iAUC comparison showed significant differences across interventions, with a notable decrease in LCEB by 1093 (SEM 438, *p* = 0.04) pmol/L·min and in LC25 by 662 (SEM 302), *p* = 0.04) pmol/L·min compared to nEB.

### Postprandial substrate oxidation and energy metabolism

**Fig. 2:**
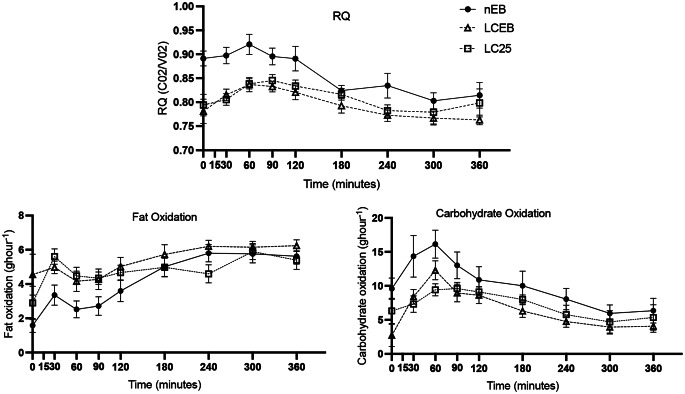
Postprandial substrate oxidation responses to liquid test meal. Data presented with standard error bars *n* 12

Postprandial responses to the liquid test meal following each intervention day are presented in Fig. 2. There was a significant main dietary effect on fat oxidation (p *<* 0.001), with lower rates in both LCEB (p *<* 0.001) and LC25 (*p* = 0.02) diets. Carbohydrate oxidation also differed significantly across diets (*p* = 0.03) with a main effect of diet, being notably lower in LCEB (*p* = 0.04) and trending lower in LC25 (*p* = 0.09). Correspondingly, respiratory quotient (RQ) levels significantly decreased in low- carbohydrate groups compared to nEB (p *<* 0.01). However, no significant differences were noted in diet-induced thermogenesis across all study arms.

### Fasting metabolism

Fasting measurements are illustrated in Table 3. Respiratory quotient (RQ) was significantly lower following both low carbohydrate (LC) diets compared to the nEB diet (LCEB vs. nEB p *<* 0.001; LC25 vs. nEB *p* = 0.02), indicating reduced fasted carbohydrate utilisation. Correspondingly, fasting plasma 3-OHB levels were notably higher in LCEB (p *<* 0.01) and trended higher in LC25 (*p* = 0.057) relative to nEB, suggestive of increased fat oxidation. No significant differences were noted in RQ and 3-OHB levels between LCEB and LC25. The LC25 diet also resulted in a significantly higher fasting GLP-1 levels compared to nEB (*p* = 0.03), with no marked difference in LCEB (*p* = 0.14). Resting energy expenditure and other fasting measurements such as glucose, TAG, insulin, and NEFA (*p* = 0.08) showed no significant differences.

**Table 3:** Fasting measurements

	nEB	LCEB	LC25
Glucose (mmol/l)	4.47 ± 0.15	4.36 ± 0.15	4.10 ± 0.12
Insulin (pmol/L)	2.74 ± 0.46	2.98 ± 0.80	2.64 ± 0.45
NEFA (mmol/l)	0.53 ± 0.07	0.86 ± 0.09	0.80 ± 0.10
TAG (mmol/l)	0.86 ± 0.09	0.81 ± 0.08	0.76 ± 0.08
3-OHB (mmol/l)	0.15 ± 0.06	0.4 ± 0.09*	0.53 ± 0.12
GLP-1 (pmol/L)	17.42 ± 2.57	25.26 ± 3.25	23.68 ± 3.39*
REE (kcal)	1602 ± 52.20	1690 ± 78.42	1655 ± 62.87
RQ (VCO_2_/VO_2_)	0.89 ± 0.016	0.78 ± 0.025*	0.79 ± 0.022*

Mean ± SEM

*significantly different from nEB, (*p* < 0.005)

Glucose, Insulin, TAG, NEFA: n 12, GLP-1:n 8

### Perceived appetite sensations and dietary intakes

Appetite sensation data of dietary intervention day indicated that hunger levels were significantly higher on the LCEB diet by 60.3% (p *<* 0.01) and the LC25 diet by 25.2% (*p* = 0.03) compared to the nEB diet. Similarly, perceived eating capacity increased by 10.4% for LCEB and 27.8% for LC25. No significant differences were observed in fullness scores (p *>* 0.05). Additionally, cravings for various tastes (sweet, savoury, salty, fatty) showed no significant differences among dietary groups (p *>* 0.05). On the subsequent metabolic study day, postprandial hunger was lower following LCEB (*p* = 0.06) and LC25 (*p* = 0.01) diets compared to nEB, by 2.4% and 8.6% respectively. However, no significant differences were found in food intake during the subsequent ad-libitum pasta meal across dietary groups (p *>* 0.05, data not presented). Over two days post-intervention, total energy and macronutrient intake showed no significant differences across diets (p *>* 0.05, data not presented). Data from five participants were excluded due to incompleteness, under-powering the analysis.

## Discussion

Existing research primarily focuses on the metabolic effects of carbohydrate restriction, either without energy restriction or with severe energy restriction in the form of fasting. Our study uniquely bridges this gap by directly comparing these dietary strategies, specifically isolating the effects of carbohydrate restriction on acute postprandial substrate and energy metabolism following food intake. We underscore the pivotal role of carbohydrate intake in modulating metabolic responses, independent of energy levels, and explore its potential to replicate the metabolic advantages associated with IER, without directly targeting weight loss. The findings provide new insights into dietary strategies showing that manipulating carbohydrate intake can achieve metabolic effects akin to acute fasting.

A key finding from our study is that LC diets elicited statistically similar metabolic responses across most measurements, including the primary outcome, postprandial TAG responses. Both LC diets achieved significant reductions in TAG levels, underscoring the role of carbohydrate restriction in attenuating postprandial lipaemia, a critical cardiovascular risk factor [4] independent of energy restriction.

Further analysis revealed similar responses in other substrate metabolites, with notable exceptions being fasting levels of 3-OHB and GLP-1, both primary indicators of ketogenesis and metabolic regulation, respectively. Notably, both LC25 and LCEB elevated 3-OHB levels, consistent with previous LCHF studies [32–34], though only LC25 reached significance. This may be due to LC25’s lower fat intake enhancing endogenous lipolysis and ketogenesis, while LCEB delivers more exogenous chylomicron-derived TAG [35, 36]. This may also be a consequence of, the overnight fasting being of insufficient duration to elevate ketone levels in LCEB, limited power due to sample size, and individual variability. Moreover, the failure to see statistically significant increases in fasting NEFA levels across both LC diets, as observed in other studies [18, 32, 33], may be due limited sample size. However, fasting levels of TAG, glucose and insulin levels remained unchanged, contradicting similar studies [18, 32, 33], potentially due to metabolically healthy participant profile. Nonetheless, upon consumption of a standard meal, chylomicron-TAG became the primary fat source [37] and hepatic 3-OHB production rose similarly in both LC diets, paralleling other lipaemia markers.

The postprandial response to the test meal further demonstrated a metabolic state similar to acute fasting, characterised by a shift favouring fatty acids and ketones over glucose as the primary energy source [38]. This shift is evidenced by decreased postprandial RQ levels, carbohydrate oxidation and increased fat oxidation, aligning with prior research [18]. Reduced glucose availability prompts the diversion of fatty acids from circulating lipoprotein-TAG, facilitated by increased lipoprotein lipase (LPL) activity in muscle, and hepatic storage towards beta-oxidation and ketogenesis [39]. Demonstrative of this mechanism, our postprandial response to test meal further demonstrated an increase in hepatic production of 3-OHB and NEFA levels, consistent with similarly designed studies [18, 34, 40]. Favourable reductions in postprandial TAG levels after LC diets, are conducive to reduced fatty acid availability for VLDL-TAG secretion and hepatic TAG re-esterification and increased chylomicron-TAG utilisation in the postprandial state due to increased fatty acid oxidation [37] as seen in comparable fasting study with LC25 arm [18]. This supports the notion that limiting carbohydrates to 50 g/day induces a lipolytic state that may continue into the next day, despite subsequent intake of carbohydrate and insulin release. This can be attributed to reduced carbohydrate intake, driven by elevated muscle LPL activity in response to limited carbohydrate availability, rather than reduced energy intake.

Improvements in the lipaemia profile coupled to higher postprandial glucose levels after both LC diets in our study, align with findings from previous LCHF [33, 34, 41] and fasting-mimicking studies [18, 40, 42]. However, unlike previous findings linking elevated glucose to decreased first-phase insulin release as a consequence of higher fasted NEFA levels [33, 34], we found no significant changes in fasted NEFA levels or in first-phase insulin secretion or other insulin sensitivity analysis. Our observed stability in postprandial insulin levels suggests that increased glucose may not always stem from altered insulin dynamics. The observed reduction in postprandial NEFA levels across LC diets likely reflects enhanced NEFA uptake in muscle, potentially due to the observed elevation in fat oxidation, or suppressed NEFA outflow from adipose tissue (i.e., lipolysis suppression), rather than insulin resistance. Further tracer studies would be required to confirm this mechanism. This is further complicated by the decreased GLP-1 levels observed after both LC diets, which is not seen in earlier research [33, 34, 40, 43]. Given GLP-1’s insulin stimulating function, alternative mechanisms could be influencing glucose regulation, warranting a need for further analysis, possibly through C-peptide measurements, to better decipher these results.

Nevertheless, studies identifying concurrent elevations in NEFA and glucose levels [18, 34, 42] align with our findings and offer insight into the mechanistic basis. This can be part of adaptive metabolism, wherein enhanced NEFA uptake and increased beta-oxidation reduce glucose uptake via the accumulation of metabolic intermediates (acetyl-CoA, NADH, citrate) [44–46]. Such intermediates interfere with carbohydrate utilisation by impeding GLUT4 translocation, inhibition of glycolysis, and lower pyruvate dehydrogenase (PDH) activity [44–46], illustrative of the intricate lipid-glucose metabolic interaction. Additionally, heightened intracellular NEFA levels can temporarily overload mitochondria, leading to dysfunction, excessive reactive oxygen species (ROS) production, and accumulation of lipotoxic intermediates, disrupting insulin signalling contributing to insulin resistance across tissues [47–49]. Numao et al. [34] attributed this elevation in NEFA and glucose levels to the high fat content of their LCHF diet; however, our research shows similar metabolic effects in LC diets both with high-fat (LCEB) and very low-fat (LC25) suggesting glucose scarcity as the primary driver. A notion supported by fasting studies [40, 42] and our group’s previous study [18], which demonstrates dose-response effects of both complete fasting and 75% energy restriction on postprandial metabolism, attributable to differences in dietary carbohydrate content. Current findings support the concept that acute metabolic challenges are indicative of short-term tachyphylaxis, (a rapid decrease in response to a metabolic stimulus), which may either attenuate or transform with prolonged dietary exposure. The observed impairment in glucose handling could, therefore, be potentially mitigated over time through metabolic adaptation which indeed is supported by studies on repeated cycles of carbohydrate restriction, both with energy restriction (e.g., IER) [14, 50] and possibly without energy restriction [51, 52]. However, both non-energy restriction studies, due to their exploratory nature, require cautious interpretation and further research. The latter study’s focus on women with breast cancer following a strict Mediterranean LC diet that may not mirror typical eating habits, affecting broader applicability and adherence in non-research settings. The former study, on the other hand, involved participants aged nine to thirty, and had varying diet restriction days between groups (intermittent LC with 7 days vs. IER with 4 days), potentially affecting outcomes. The longer carbohydrate restriction in the intermittent LC group might lead to more pronounced metabolic adaptations, such as increased fat oxidation or changes in ketone body levels and impact adherence and gut microbiota, complicating dietary approach comparisons.

The enhancement in fat oxidation from a one-day low-carbohydrate diet, irrespective of energy content, and the response to reintroduced carbohydrates can be seen as demonstrative of metabolic flexibility [53]. Augmented uptake of NEFAs by peripheral tissue, enhances fatty acid oxidation, minimises TAG accumulation, and improves insulin signalling in skeletal muscle, potentially offering significant health advantages [54]. A recent study by Antoni et al. [14] has shown that IER, inherently low in carbohydrate, leads to better postprandial lipid responses than CER with equal caloric intake and matched weight loss. This suggests that IER enhances metabolic flexibility by regularly activating a fat oxidation state. In contrast, benefits of traditional, CER’s benefits primarily stem from weight loss, which usually lacks a substantial shift to fat oxidation. Similarly, another study [55] highlights metabolic improvements in IER without weight loss, underlining the significance of alternating fuel oxidation in dietary approaches. Coupling these observations with our current study, it could be hypothesised that cycling between low-carbohydrate and regular carbohydrate intake might amplify this flexibility, thus improving metabolic health independently of weight loss [16].

Also, it is important to note that in terms of practical application, the primary motivation for adopting LC diets is often for weight loss [56], which by itself brings metabolic benefits [57]. A secondary objective of our study was to explore potential energy balance effects. The LCEB arm also was proposed to increase compliance by reducing hunger and increasing food availability, especially important if long-term repeated cycles of LCEB are adopted. Interestingly, in the appetite sensations while participants felt increased hunger on restriction days, consistent with prior fasting studies [40, 42], the hunger decreased after a test meal in both LC diet (i.e. with or without an acute energy deficit), potentially correlating with higher ketone levels as also evidenced by VLCD and ketogenic diets [58]. Moreover, variations in hunger did not translate into subsequent food intake as there was no difference in 48-hour *ad libitum* food intake for either diet. This indicated that the LC25 group maintained a 75% energy deficit with little compensation in intake, consistent with prior research [18]. Moreover, real-life adherence in the long-term might differ, low carbohydrate diets tend to reduce spontaneous food intake over time [59], suggesting that long-term energy intake under LCEB diets might still lead to an overall energy deficit, underscoring the need for further studies.

With regard to energy expenditure our study found no significant changes in short term REE or DIT consistent with findings from short-term energy-restricted diets [18, 40, 42]. While some literature indicates an acute increase in energy expenditure with low-carbohydrate and fasting diets due to heightened gluconeogenesis, protein turnover, TAG-fatty acid cycling, and ketogenesis [20, 60], long-term outcomes remain mixed. Doubly labelled water studies report variable effects on energy expenditure (EE) in prolonged low-carbohydrate diets, with some indicating increases [61, 62] and others showing no change [63]. This variation could stem from inaccuracies in RQ estimation, which might overestimate EE values when based solely on VCO2 [64]. In contrast, IER studies typically note adaptive decreases in REE [14, 65], suggesting different metabolic adaptations. It should be noted, however, that despite varying findings on energy expenditure, it’s crucial to recognize that these changes are likely too minor to significantly impact energy balance through adaptive changes in REE.

The main strength of the study was the novel comparisons between two low carbohydrate diets at different energy levels and two energy-balanced diets at different carbohydrate contents, from both a metabolic and energy balance perspective. The crossover design is a strength due to minimising individual variability. Yet we acknowledge that our study was limited to only examining postprandial responses following a single test meal, which may not fully capture the variability in postprandial metabolism related to meal composition and timing. We also acknowledge the limited sample size and metabolically healthy profile of our cohort which may explain why some postprandial results between LCEB and nEB showed only a trend, without statistical significance. A larger or metabolically compromised group might have yielded more pronounced differences.

### Summary and future direction

In summary, our study demonstrated that carbohydrate restriction, both with and without energy restriction induces short-term changes in fasted and postprandial glucose and lipid metabolism, along with similar effects on appetite measurements and subsequent energy compensation in healthy overweight/obese adults. The lack of fasted and postprandial differences between our low-carbohydrate intervention arms raises critical questions but suggest a dominant role of carbohydrate restriction itself in inducing metabolic adaptations, potentially overshadowing the differences due to energy content. Therefore, the metabolic threshold for inducing these adaptations might be more achievable, than previously thought. Further research is warranted to investigate how metabolism and behaviours adapt to repetitive cycles of carbohydrate restriction at different energy levels and whether the observed results could replicate the impact of intermittent energy-restricted dietary regimens.

## Electronic supplementary material

Below is the link to the electronic supplementary material.

Supplementary Material 1

## References

[1] (WHO) WHO (2022) WHO European regional obesity report 2022. Copenhagen

[2] Kelly T, Yang W, Chen C-S, Reynolds K, He J (2008) Global burden of obesity in 2005 and projections to 2030. Int J Obes 32(9):1431–1437 10.1038/ijo.2008.102 18607383

[3] Jehan S, Zizi F, Pandi-Perumal SR, McFarlane SI, Jean-Louis G, Myers AK (2020) Energy imbalance: obesity, associated comorbidities, prevention, management and public health implications. Advances in obesity. Weight Manage Control 10(5):146 PMC7725222 33305001

[4] Ding C, Chan Z, Magkos F (2016) Lean, but not healthy: the ‘metabolically obese, normal-weight’phenotype. Curr Opin Clin Nutr Metabolic Care 19(6):408–417 10.1097/MCO.0000000000000317 27552473

[5] van der A DL, Nooyens AC, van Duijnhoven FJ, Verschuren MM, Boer JM (2014) All-cause mortality risk of metabolically healthy abdominal obese individuals: the EPIC‐MORGEN study. Obesity 22(2):557–564 23595997 10.1002/oby.20480

[6] Varady KA, Cienfuegos S, Ezpeleta M, Gabel K (2022) Clinical application of intermittent fasting for weight loss: progress and future directions. Nat Reviews Endocrinol 18(5):309–321 10.1038/s41574-022-00638-x 35194176

[7] Harris L, Hamilton S, Azevedo LB, Olajide J, De Brún C, Waller G et al (2018) Intermittent fasting interventions for treatment of overweight and obesity in adults: a systematic review and meta-analysis. JBI Evid Synthesis 16(2):507–547 10.11124/JBISRIR-2016-003248 29419624

[8] Kim K-K, Kang J-H, Kim EM (2022) Updated meta-analysis of studies from 2011 to 2021 comparing the effectiveness of intermittent energy restriction and continuous energy restriction. J Obes Metabolic Syndrome 31(3):230 10.7570/jomes22050 PMC9579470 36177730

[9] Cioffi I, Evangelista A, Ponzo V, Ciccone G, Soldati L, Santarpia L et al (2018) Intermittent versus continuous energy restriction on weight loss and cardiometabolic outcomes: a systematic review and meta-analysis of randomized controlled trials. J Translational Med 16:1–15 10.1186/s12967-018-1748-4 PMC6304782 30583725

[10] Ye Y-F, Zhang M-X, Lin Z, Tang L (2022) Is intermittent fasting better than continuous energy restriction for adults with overweight and obesity? Diabetes, metabolic syndrome and obesity: targets and therapy.:2813–2826 10.2147/DMSO.S376409 PMC9484493 36134390

[11] Schwingshackl L, Zähringer J, Nitschke K, Torbahn G, Lohner S, Kühn T et al (2021) Impact of intermittent energy restriction on anthropometric outcomes and intermediate disease markers in patients with overweight and obesity: systematic review and meta-analyses. Crit Rev Food Sci Nutr 61(8):1293–1304 32363896 10.1080/10408398.2020.1757616

[12] He S, Wang J, Zhang J, Xu J (2021) Intermittent versus continuous energy restriction for weight loss and metabolic improvement: a meta-analysis and systematic review. Obesity 29(1):108–115 34494373 10.1002/oby.23023

[13] Wing RR, Lang W, Wadden TA, Safford M, Knowler WC, Bertoni AG et al (2011) Benefits of modest weight loss in improving cardiovascular risk factors in overweight and obese individuals with type 2 diabetes. Diabetes Care 34(7):1481–1486 21593294 10.2337/dc10-2415 PMC3120182

[14] Antoni R, Johnston KL, Collins AL, Robertson MD (2018) Intermittent V. continuous energy restriction: differential effects on postprandial glucose and lipid metabolism following matched weight loss in overweight/obese participants. Br J Nutr 119(5):507–516 29508693 10.1017/S0007114517003890

[15] De Cabo R, Mattson MP (2019) Effects of intermittent fasting on health, aging, and disease. N Engl J Med 381(26):2541–2551 31881139 10.1056/NEJMra1905136

[16] Goodpaster BH, Sparks LM (2017) Metabolic flexibility in health and disease. Cell Metabol 25(5):1027–1036 10.1016/j.cmet.2017.04.015 PMC5513193 28467922

[17] Klein S, Wolfe R (1992) Carbohydrate restriction regulates the adaptive response to fasting. Am J Physiology-Endocrinology Metabolism 262(5):E631–E6 10.1152/ajpendo.1992.262.5.E631 1590373

[18] Antoni R, Johnston KL, Collins AL, Robertson MD (2016) Investigation into the acute effects of total and partial energy restriction on postprandial metabolism among overweight/obese participants. Br J Nutr 115(6):951–959 26819200 10.1017/S0007114515005346

[19] Rietman A, Schwarz J, Tomé D, Kok FJ, Mensink M (2014) High dietary protein intake, reducing or eliciting insulin resistance? Eur J Clin Nutr 68(9):973–979 24986822 10.1038/ejcn.2014.123

[20] Fine EJ, Feinman RD (2004) Thermodynamics of weight loss diets. Nutr Metabolism 1:1–8 10.1186/1743-7075-1-15 PMC543577 15588283

[21] Team NS (2016) Government dietary recommendations: government recommendations for energy and nutrients for males and females aged 1–18 years and 19 + years. Public Health Engl

[22] Matsuda M, DeFronzo RA (1999) Insulin sensitivity indices obtained from oral glucose tolerance testing: comparison with the euglycemic insulin clamp. Diabetes Care 22(9):1462–1470 10480510 10.2337/diacare.22.9.1462

[23] Matthews DR, Hosker JP, Rudenski AS, Naylor B, Treacher DF, Turner R (1985) Homeostasis model assessment: insulin resistance and β-cell function from fasting plasma glucose and insulin concentrations in man. Diabetologia 28:412–419 3899825 10.1007/BF00280883

[24] Tura A, Kautzky-Willer A, Pacini G (2006) Insulinogenic indices from insulin and C-peptide: comparison of beta-cell function from OGTT and IVGTT. Diabetes Res Clin Pract 72(3):298–301 16325298 10.1016/j.diabres.2005.10.005

[25] Retnakaran R, Qi Y, Goran M, Hamilton J (2009) Evaluation of proposed oral disposition index measures in relation to the actual disposition index. Diabet Med 26(12):1198–1203 20002470 10.1111/j.1464-5491.2009.02841.x

[26] Stumvoll M, Mitrakou A, Pimenta W, Jenssen T, Yki-Järvinen H, Van Haeften T et al (2000) Use of the oral glucose tolerance test to assess insulin release and insulin sensitivity. Diabetes Care 23(3):295–301 10868854 10.2337/diacare.23.3.295

[27] Weir JdV (1949) New methods for calculating metabolic rate with special reference to protein metabolism. J Physiol 109(1–2):1 15394301 10.1113/jphysiol.1949.sp004363 PMC1392602

[28] Frayn K (1983) Calculation of substrate oxidation rates in vivo from gaseous exchange. J Appl Physiol 55(2):628–634 6618956 10.1152/jappl.1983.55.2.628

[29] Westerterp KR (2004) Diet induced thermogenesis. Nutr Metabolism 1:1–5 10.1186/1743-7075-1-5 PMC524030 15507147

[30] Stubbs RJ, Hughes DA, Johnstone AM, Rowley E, Reid C, Elia M et al (2000) The use of visual analogue scales to assess motivation to eat in human subjects: a review of their reliability and validity with an evaluation of new hand-held computerized systems for Temporal tracking of appetite ratings. Br J Nutr 84(4):405–415 11103211 10.1017/s0007114500001719

[31] Floch J-PL, Escuyer P, Baudin E, Baudon D, Perlemuter L (1990) Blood glucose area under the curve: methodological aspects. Diabetes Care 13(2):172–175 2351014 10.2337/diacare.13.2.172

[32] Hengist A, Davies R, Rogers P, Brunstrom J, Van Loon L, Walhin J-P et al (2022) Restricting sugar or carbohydrate intake does not impact physical activity level or energy intake over 24 hours despite changes in substrate use: A randomised crossover. Curr Developments Nutr 6:444 10.1007/s00394-022-03048-x PMC9941259 36326863

[33] Numao S, Kawano H, Endo N, Yamada Y, Konishi M, Takahashi M et al (2012) Short-term low carbohydrate/high-fat diet intake increases postprandial plasma glucose and glucagon-like peptide-1 levels during an oral glucose tolerance test in healthy men. Eur J Clin Nutr 66(8):926–931 22669333 10.1038/ejcn.2012.58

[34] Numao S, Kawano H, Endo N, Yamada Y, Takahashi M, Konishi M et al (2016) Short-term high-fat diters postprandial glucose metabolism and circulating vascular cell adhesion molecule-1 in healthy males. Applied Physiology, Nutrition, and Metabolism.;41(8):895–902 10.1139/apnm-2015-0702 27454856

[35] Kiens B, Essen-Gustavsson B, Gad P, Lithell H (1987) Lipoprotein lipase activity and intramuscular triglyceride stores after long‐term high‐fat and high‐carbohydrate diets in physically trained men. Clin Physiol 7(1):1–9 3545651 10.1111/j.1475-097x.1987.tb00628.x

[36] Schrauwen P, Wagenmakers A, van Marken Lichtenbelt WD, Saris W, Westerterp KR (2000) Increase in fat oxidation on a high-fat diet is accompanied by an increase in triglyceride-derived fatty acid oxidation. Diabetes 49(4):640–646 10871203 10.2337/diabetes.49.4.640

[37] Karpe F, Hellénius M-L, Hamsten A (1999) Differences in postprandial concentrations of very—low-density lipoprotein and chylomicron remnants between normotriglyceridemic and hypertriglyceridemic men with and without coronary heart disease. Metabolism 48(3):301–307 10094104 10.1016/s0026-0495(99)90076-8

[38] Westman EC, Mavropoulos J, Yancy WS, Volek JS (2003) A review of low-carbohydrate ketogenic diets. Curr Atheroscler Rep 5:476–483 14525681 10.1007/s11883-003-0038-6

[39] Plaisance EP, Fisher G (2014) Exercise and dietary-mediated reductions in postprandial lipemia. J Nutr Metabolism 2014(1):902065 10.1155/2014/902065 PMC4100364 25061524

[40] Clayton DJ, Creese M, Skidmore N, Stensel DJ, James LJ (2016) No effect of 24 h severe energy restriction on appetite regulation and ad libitum energy intake in overweight and obese males. Int J Obes 40(11):1662–1670 10.1038/ijo.2016.106 27339607

[41] Kanamori K, Ihana-Sugiyama N, Yamamoto-Honda R, Nakamura T, Sobe C, Kamiya S et al (2017) Postprandial glucose surges after extremely low carbohydrate diet in healthy adults. Tohoku J Exp Med 243(1):35–39 28924074 10.1620/tjem.243.35

[42] Clayton DJ, Burrell K, Mynott G, Creese M, Skidmore N, Stensel DJ et al (2016) Effect of 24-h severe energy restriction on appetite regulation and ad libitum energy intake in lean men and women. Am J Clin Nutr 104(6):1545–1553 27806971 10.3945/ajcn.116.136937

[43] Van Nierop FS, Meessen EC, Nelissen KG, Achterbergh R, Lammers LA, Vaz FM et al (2019) Differential effects of a 40-hour fast and bile acid supplementation on human GLP-1 and FGF19 responses. Am J Physiology-Endocrinology Metabolism 317(3):E494–E502 10.1152/ajpendo.00534.2018 31237451

[44] Roden M, Price TB, Perseghin G, Petersen KF, Rothman DL, Cline GW et al (1996) Mechanism of free fatty acid-induced insulin resistance in humans. J Clin Investig 97(12):2859–2865 8675698 10.1172/JCI118742 PMC507380

[45] Randle P, Garland P, Hales C, Newsholme E (eds) (1964) The glucose fatty acid cycle and diabetes mellitus. Ciba Foundation Symposium-Aetiology of Diabetes Mellitus and its Complications (Colloquia on Endocrinology); Wiley Online Library

[46] Boden G (2002) Interaction between free fatty acids and glucose metabolism. Curr Opin Clin Nutr Metabolic Care 5(5):545–549 10.1097/00075197-200209000-00014 12172479

[47] Poitout V, Robertson RP (2008) Glucolipotoxicity: fuel excess and β-cell dysfunction. Endocr Rev 29(3):351–366 18048763 10.1210/er.2007-0023 PMC2528858

[48] Kim J-a, Wei Y, Sowers JR (2008) Role of mitochondrial dysfunction in insulin resistance. Circul Res 102(4):401–414 10.1161/CIRCRESAHA.107.165472 PMC2963150 18309108

[49] Holland WL, Brozinick JT, Wang L-P, Hawkins ED, Sargent KM, Liu Y et al (2007) Inhibition of ceramide synthesis ameliorates glucocorticoid-, saturated-fat-, and obesity-induced insulin resistance. Cell Metabol 5(3):167–179 10.1016/j.cmet.2007.01.002 17339025

[50] Gao Y, Tsintzas K, Macdonald IA, Cordon SM, Taylor MA (2022) Effects of intermittent (5: 2) or continuous energy restriction on basal and postprandial metabolism: A randomised study in normal-weight, young participants. Eur J Clin Nutr 76(1):65–73 34040199 10.1038/s41430-021-00909-2 PMC8766278

[51] Dou Y, Jiang Y, Chen X, Zhang Y, Wang Y, Chen H et al (2023) Intermittent dietary carbohydrate restriction versus calorie restriction and cardiometabolic profiles: A randomized trial. Obesity 31(9):2260–2271 37545298 10.1002/oby.23855

[52] Harvie M, Wright C, Pegington M, McMullan D, Mitchell E, Martin B et al (2013) The effect of intermittent energy and carbohydrate restriction V. daily energy restriction on weight loss and metabolic disease risk markers in overweight women. Br J Nutr 110(8):1534–1547 23591120 10.1017/S0007114513000792 PMC5857384

[53] Galgani JE, Moro C, Ravussin E (2008) Metabolic flexibility and insulin resistance. Am J physiology-endocrinology Metabolism 295(5):E1009–E17 10.1152/ajpendo.90558.2008 PMC2584808 18765680

[54] Corpeleijn E, Saris WH, Blaak EE (2009) Metabolic flexibility in the development of insulin resistance and type 2 diabetes: effects of lifestyle. Obes Rev 10(2):178–193 19207879 10.1111/j.1467-789X.2008.00544.x

[55] Sundfør T, Svendsen M, Tonstad S (2018) Effect of intermittent versus continuous energy restriction on weight loss, maintenance and cardiometabolic risk: A randomized 1-year trial. Nutr Metabolism Cardiovasc Dis 28(7):698–706 10.1016/j.numecd.2018.03.009 29778565

[56] Churuangsuk C, Lean ME, Combet E (2020) Carbohydrate knowledge, dietary guideline awareness, motivations and beliefs underlying low-carbohydrate dietary behaviours. Sci Rep 10(1):14423 32879368 10.1038/s41598-020-70905-2 PMC7468104

[57] Van Gaal L, Wauters M, De Leeuw I (1997) The beneficial effects of modest weight loss on cardiovascular risk factors. Int J Obes Relat Metabolic Disorders: J Int Association Study Obes 21:S5–9 9130034

[58] Gibson AA, Seimon RV, Lee CM, Ayre J, Franklin J, Markovic T et al (2015) Do ketogenic diets really suppress appetite? A systematic review and meta-analysis. Obes Rev 16(1):64–76 25402637 10.1111/obr.12230

[59] Westman EC, Feinman RD, Mavropoulos JC, Vernon MC, Volek JS, Wortman JA et al (2007) Low-carbohydrate nutrition and metabolism. Am J Clin Nutr 86(2):276–284 17684196 10.1093/ajcn/86.2.276

[60] Basolo A, Magno S, Santini F, Ceccarini G (2022) Ketogenic diet and weight loss: is there an effect on energy expenditure? Nutrients 14(9):1814 35565778 10.3390/nu14091814 PMC9105638

[61] Ebbeling CB, Feldman HA, Klein GL, Wong JM, Bielak L, Steltz SK et al (2018) Effects of a low carbohydrate diet on energy expenditure during weight loss maintenance: randomized trial. BMJ.;363 10.1136/bmj.k4583 PMC6233655 30429127

[62] Ebbeling CB, Swain JF, Feldman HA, Wong WW, Hachey DL, Garcia-Lago E et al (2012) Effects of dietary composition during weight loss maintenance: a controlled feeding study. JAMA: J Am Med Association 307(24):2627 10.1001/jama.2012.6607 PMC3564212 22735432

[63] Hengist A, Davies RG, Walhin J-P, Buniam J, Merrell LH, Rogers L et al (2024) Ketogenic diet but not free-sugar restriction alters glucose tolerance, lipid metabolism, peripheral tissue phenotype, and gut microbiome: RCT. Cell Rep Med.;5(8) 10.1016/j.xcrm.2024.101667 PMC11384946 39106867

[64] Hall KD, Guo J, Chen KY, Leibel RL, Reitman ML, Rosenbaum M et al (2019) Methodologic considerations for measuring energy expenditure differences between diets varying in carbohydrate using the doubly labeled water method. Am J Clin Nutr 109(5):1328–1334 31028699 10.1093/ajcn/nqy390 PMC6499509

[65] Byrne NM, Sainsbury A, King NA, Hills AP, Wood R (2018) Intermittent energy restriction improves weight loss efficiency in obese men: the MATADOR study. Int J Obes 42(2):129–138 10.1038/ijo.2017.206 PMC5803575 28925405

