# Supplementary figures and images for: Isolating the acute metabolic effects of carbohydrate restriction on postprandial metabolism with or without energy restriction: a crossover study
Source: Eur J Nutr. 2025 Mar 20;64(3):133. doi: 10.1007/s00394-025-03646-5 (PMC11926029; doi:10.1007/s00394-025-03646-5)

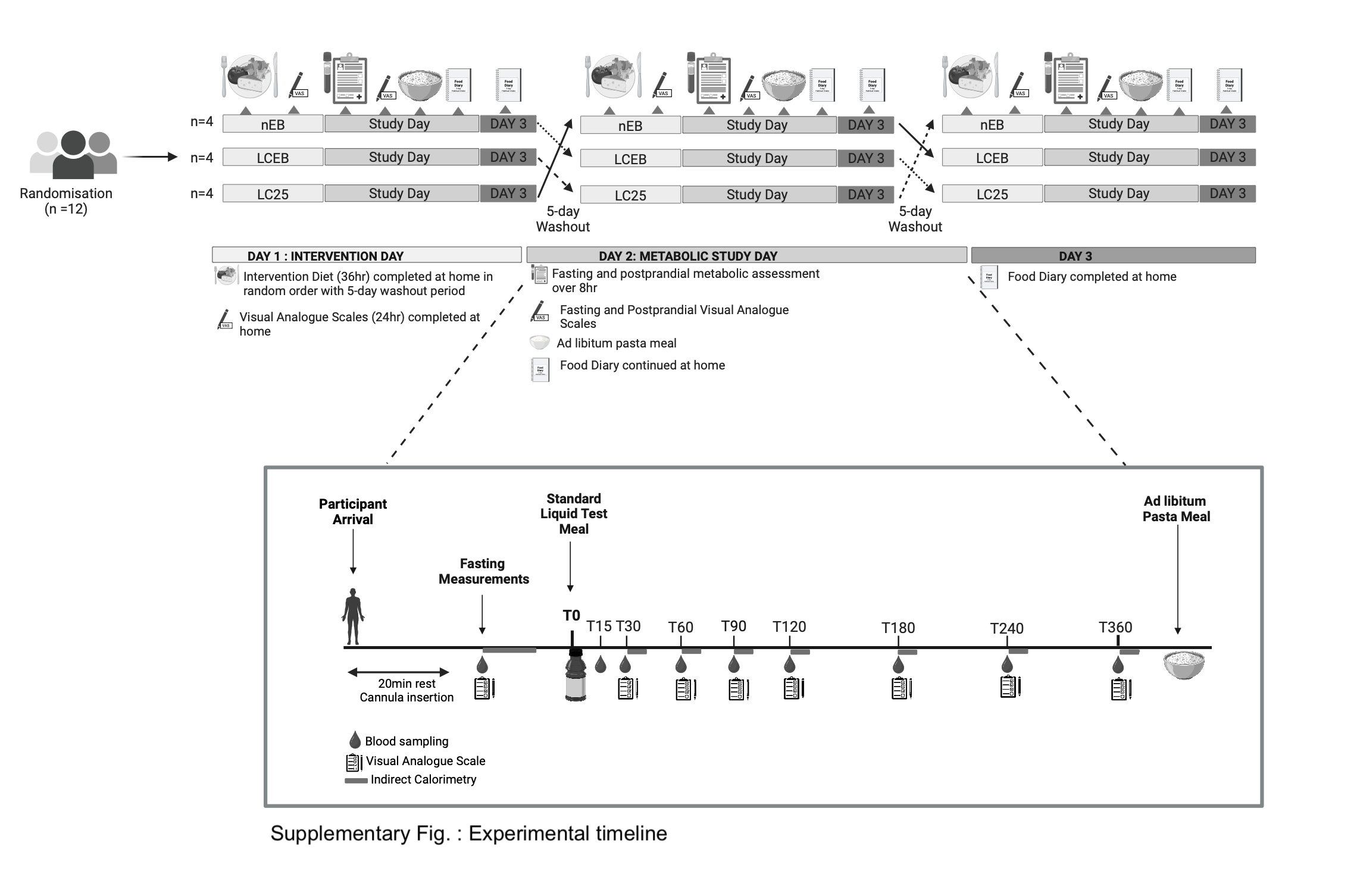

Supplement: Supplementary file 1 — Supplementary Material 1 [file 394_2025_3646_MOESM1_ESM.png]
